# Transcriptional Analysis of Infection With Early or Late Isolates From the 2013–2016 West Africa Ebola Virus Epidemic Does Not Suggest Attenuated Pathogenicity as a Result of Genetic Variation

**DOI:** 10.3389/fmicb.2021.714817

**Published:** 2021-08-13

**Authors:** Kevin J. Maroney, Amanda N. Pinski, Andrea Marzi, Ilhem Messaoudi

**Affiliations:** ^1^Department of Molecular Biology and Biochemistry, University of California, Irvine, Irvine, CA, United States; ^2^Laboratory of Virology, Division of Intramural Research, National Institute of Allergy and Infectious Diseases, NIH, Rocky Mountain Laboratories, Hamilton, MT, United States; ^3^Center for Virus Research, University of California, Irvine, Irvine, CA, United States; ^4^Institute for Immunology, University of California, Irvine, Irvine, CA, United States

**Keywords:** Ebola virus, transcriptomic (RNA-Seq), viral epidemiology, nonhuman primate (macaque), Ebola virus disease

## Abstract

The 2013–2016 West Africa Ebola virus (EBOV) epidemic caused by the EBOV-Makona isolate is the largest and longest recorded to date. It incurred over 28,000 infections and ∼11,000 deaths. Early in this epidemic, several mutations in viral glycoprotein (A82V), nucleoprotein (R111C), and polymerase L (D759G) emerged and stabilized. *In vitro* studies of these new EBOV-Makona isolates showed enhanced fitness and viral replication capacity. However, *in vivo* studies in mice and rhesus macaques did not provide any evidence of enhanced viral fitness or shedding. Infection with late isolates carrying or early isolates lacking (early) these mutations resulted in uniformly lethal disease in nonhuman primates (NHPs), albeit with slightly delayed kinetics with late isolates. The recent report of a possible reemergence of EBOV from a persistent infection in a survivor of the epidemic highlights the urgency for understanding the impact of genetic variation on EBOV pathogenesis. However, potential molecular differences in host responses remain unknown. To address this gap in knowledge, we conducted the first comparative analysis of the host responses to lethal infection with EBOV-Mayinga and EBOV-Makona isolates using bivariate, longitudinal, regression, and discrimination transcriptomic analyses. Our analysis shows a conserved core of differentially expressed genes (DEGs) involved in antiviral defense, immune cell activation, and inflammatory processes in response to EBOV-Makona and EBOV-Mayinga infections. Additionally, EBOV-Makona and EBOV-Mayinga infections could be discriminated based on the expression pattern of a small subset of genes. Transcriptional responses to EBOV-Makona isolates that emerged later during the epidemic, specifically those from Mali and Liberia, lacked signatures of profound lymphopenia and excessive inflammation seen following infection with EBOV-Mayinga and early EBOV-Makona isolate C07. Overall, these findings provide novel insight into the mechanisms underlying the lower case fatality rate (CFR) observed with EBOV-Makona compared to EBOV-Mayinga.

## Introduction

Zaire Ebola virus (EBOV) is a single-stranded, negative-sense RNA virus and a member of the *Filoviridae* family that is responsible for Ebola virus disease (EVD; Sanchez et al., 1993; Bell et al., 2015). EVD is characterized by lymphopenia, excessive inflammation, thrombocytopenia, and disseminated intravascular coagulation, ultimately leading to multi-organ failure and a high case fatality rate (CFR, 40–90%) (Geisbert et al., 2003b; Feldmann and Geisbert, 2011). EBOV preferentially infects antigen presenting cells (APCs), notably monocytes, macrophages, and dendritic cells (DCs), that play a critical role in viral dissemination (Geisbert et al., 2003a; Menicucci et al., 2017). Moreover, infected monocytes are a major source of pro-inflammatory cytokines, which precipitate the development of coagulopathy and organ injury (Geisbert et al., 2000, 2003b; Gupta et al., 2001). In contrast, EBOV infection of DCs interferes with their maturation and ability to mobilize T cells, thwarting the development of cellular and humoral immunity (Marzi et al., 2013; Barrenas et al., 2015; Iampietro et al., 2017; Pleet et al., 2017, 2018).

Different strains of EBOV show a considerable diversity in pathogenicity, lethality, and disease progression rate despite sharing ∼97% genome similarity (Bell et al., 2015; Marzi et al., 2015, 2018; Wong et al., 2016; Menicucci et al., 2017; Versteeg et al., 2017, 2019; Madelain et al., 2018; McMullan et al., 2019; Pinski and Messaoudi, 2020). The 1976 outbreak of EBOV-Mayinga and the 1995 outbreak of EBOV-Kikwit resulted in ∼300 cases each and CFRs of ∼90% (Centers for Disease Control and Prevention, 1995a, b; Muyembe-Tamfum et al., 1999; Kuhn et al., 2013; Kaner and Schaack, 2016). In contrast, the 2013–2016 West African epidemic caused by the EBOV-Makona isolate resulted in over 28,000 reported cases and a CFR of ∼39% (Kaner and Schaack, 2016; Shoman et al., 2017). Furthermore, EBOV-Makona accumulated nonsynonymous mutations in the nucleoprotein (NP, R111C), glycoprotein (GP, A82V), and polymerase (L, D759G) that were associated with increased replication capacity and enhanced viral fitness *in vitro* that may explain accelerated transmission (Dietzel et al., 2017; Hoffmann et al., 2017; Ruedas et al., 2017, 2018; Ueda et al., 2017; Marzi et al., 2018; Wong et al., 2019). Specifically, mutations in GP, the viral receptor for entry into host cells, could enhance infectivity. However, studies in nonhuman primates (NHP), the gold standard animal model to study EBOV pathogenesis, showed comparable clinical disease, although infection with EBOV-Makona isolates that emerged late in the epidemic, as a group (Mali and Liberia), resulted in delayed time to euthanasia (Geisbert et al., 2003a, 2015; Marzi et al., 2018; Longet et al., 2020).

Recent studies have reported distinct transcriptional responses in NHPs infected with EBOV-Makona Guinea C07 (early isolate) or EBOV-Kikwit (Rivera and Messaoudi, 2016; Versteeg et al., 2019; Pinski et al., 2021a). However, comparison of the host transcriptional responses to early and late EBOV-Makona isolates and to the historical and highly lethal EBOV-Mayinga strain are absent. Understanding similarities and differences in host responses to EBOV isolates is essential for connecting changes in pathogenicity to viral evolution, and aiding the development of vaccines and antivirals to prevent and address future outbreaks. For instance, a 2021 outbreak is believed to be caused by the reemergence of persistent EBOV in a recovered survivor of the 2013–2016 epidemic (Centers for Disease Control and Prevention, 2021a). Transcriptional studies have proven instrumental in understanding the mechanisms of pathogenesis of filoviruses (Eisfeld et al., 2017; Menicucci et al., 2017, 2019; Versteeg et al., 2017; Kotliar et al., 2020; Baillet et al., 2021; Pinski et al., 2021a, b). Therefore, in this study, we leveraged access to historical RNA samples from a previous study (Marzi et al., 2018) that compared clinical parameters following infection with early and late EBOV-Makona isolates to address this question. Specifically, we compared the transcriptional response following infection with early and late EBOV-Makona isolates as well as EBOV-Mayinga in rhesus macaques. We report that infection with the different EBOV isolates induces a transcriptional response characterized by a shared core of genes that play a role in antiviral defense, inflammation, and cell activation, which reflects EVD pathology. Additionally, we identified 300 genes involved in host defense and the stress responses that were sufficient to distinguish EBOV infections from each other during peak disease. Finally, infection with late EBOV-Makona isolates lacked transcriptional indicators of lymphopenia and robust upregulation of genes that play a role in inflammation, which may in part explain the lower CFR observed during the 2013–2016 West African epidemic.

## Materials and Methods

### Cohorts and Study Design

Historical RNA samples collected from our previous study were used in this study (Marzi et al., 2018). In this previous study, four cohorts of rhesus macaques were infected intramuscularly with 1 × 10^3^ focus forming units (FFUs) of EBOV-Mayinga (*n* = 5) or EBOV-Makona isolates: Guinea C07, Mali, or Liberia (*n* = 3 each). Whole blood (WB) samples were collected at 0, 2, 4, and 6 days post infection (DPI), and RNA was isolated from the WB of EBOV-Mayinga- and EBOV-Makona-infected NHPs using the QIAmp Viral RNA Kit (Qiagen) as previously described (Marzi et al., 2018). This prior work was performed in the maximum containment laboratory at the Rocky Mountain Laboratories (RML), Division of Intramural Research, National Institute of Allergy and Infectious Diseases, National Institutes of Health. RML is an AAALAC accredited institution. All procedures followed standard operating procedures (SOPs) approved by the RML Institutional Biosafety Committee (IBC). Animal work was performed in strict accordance with the recommendations described in the Guide for the Care and Use of Laboratory Animals of the National Institute of Health, the Office of Animal Welfare and the Animal Welfare Act, United States Department of Agriculture. The study was approved by the RML Animal Care and Use Committee (ACUC). Procedures were conducted in animals anesthetized by trained personnel under the supervision of veterinary staff. The humane endpoint criteria for euthanasia were specified and approved by the RML ACUC. All efforts were made to ameliorate animal welfare and minimize animal suffering in accordance with the Weatherall report on the use of NHPs in research^1^.

### Library Preparation and Sequencing

RNA samples from DPI 0, 4, and 6 were used in the current study. Integrity and concentration of historical RNA samples were validated on the Agilent 2100 Bioanalyzer prior to library construction. rRNA was depleted from the samples using the NEBNext rRNA Depletion kit before cDNA library construction with the NEBNext Ultra II Directional RNA Library Prep Kit (Illumina). cDNA library quality and concentration were confirmed on the Agilent 2100 Bioanalyzer before sequencing on the Illumina HiSeq2500 or NovaSeq platforms.

### Downstream Analysis and Bioinformatics

Raw sequences were trimmed to a minimum length of 75 bp and average Phred score of 30 using Trim Galore before alignment to the *Macaca mulatta* genome “Macaca_mulatta.Mmul_8.0.1.dna.toplevel.fa” using tophat. Genes were annotated with the Ensembl annotation files for *M. mulatta* (Macaca_mulatta.Mmul_8.0.1.97.gtf). Preliminary processing of RNA-Seq data was performed using the systemPipeR package available from Bioconductor (Tw and Girke, 2016).

Three approaches were used to identify differentially expressed genes (DEGs): (1) EdgeR, (2) STEM, and (3) MaSigPro (Ernst and Bar-Joseph, 2006; Robinson et al., 2010; Nueda et al., 2014). EdgeR package, which uses the Trimmed means of M-values (TMM), was used to identify DEGs, and counts were normalized using the reads per kilobase of exon per million (rpkm) (Mortazavi et al., 2008; Menicucci et al., 2017; Versteeg et al., 2019). DEGs were then filtered for only those encoding human protein-coding homologs with an average rpkm ≥ 5, FDR ≤ 0.05, and fold change ≤ −1 or ≥ 1. To identify longitudinal patterns of gene expression changes throughout infection, we used Short Time Series Expression Miner (STEM) software, which clusters genes both by significance and expression patterns (Ernst and Bar-Joseph, 2006). MaSigPro was used for regression analysis with a two-way forward regression strategy comparing the EBOV-Makona Mali, Liberia, and EBOV-Mayinga isolates to EBOV-Makona Guinea C07 (Conesa et al., 2006). Genes that contributed significantly to the most degree of variance in at least 16 different comparisons were fit into the model and were then clustered by temporal expression pattern. Sparse partial least squares discrimination analysis (sPLS-DA) was performed with the mixOmics R package for validation and classification (Rohart et al., 2017). Models were initially built with a minimum of 10 components and validated with five-fold cross-validation and 10 repetitions.

Functional enrichment was carried out using Metascape to identify gene ontology (GO) terms representing biological processes (Zhou et al., 2019). GO network plot was created with Cytoscape visualization software (Otasek et al., 2019; Zhou et al., 2019). All heatmaps, bubbleplots, and Venn diagrams were made and analyzed through R packages (R Core Team, 2015; Wickham, 2009). Digital cell quantification (DCQ) was performed using ImmQuant, a platform that uses a deconvolution algorithm to compare transcriptomic sequencing data to reference datasets in order to predict changes in cell frequencies (Abbas et al., 2005; Novershtern et al., 2011; Frishberg et al., 2016). Both DMAP and IRIS datasets were used.

### Statistical Analysis

All statistical analyses were performed in GraphPad Prism (version 7). Genes with similar temporal expression profiles were determined using time course data (analyzed through STEM) and were grouped into clusters (analysis of clusters shown in Figures 2, 4). All clusters for each isolate were regenerated on one plot with multiple biological replicates of the DEGs found through STEM (Figures 2A, 4A). ImmQuant deconvolution data was analyzed using a repeated measure mixed effect model statistical design against 0 DPI, where numbers of samples were not equal between timepoints. Specifically, multiple comparison analyses were performed between subsequent days post infection (4 DPI and 6 DPI) and baseline (0 DPI) and indicated with asterisks (Figures 3, 6 and Supplementary Figure 3). For EBOV-Makona Mali and Liberia datasets, significance was additionally tested using a two-tailed T test for population changes between 0 DPI and both 4 + 6 DPI.

### Data Availability

Sequencing data for rhesus macaques is available at BioProject PRJNA718880.

## Results

### Host Transcriptional Response to EBOV-Mayinga Highlights Dysregulation of Innate and Adaptive Immune Responses

**FIGURE 1:**
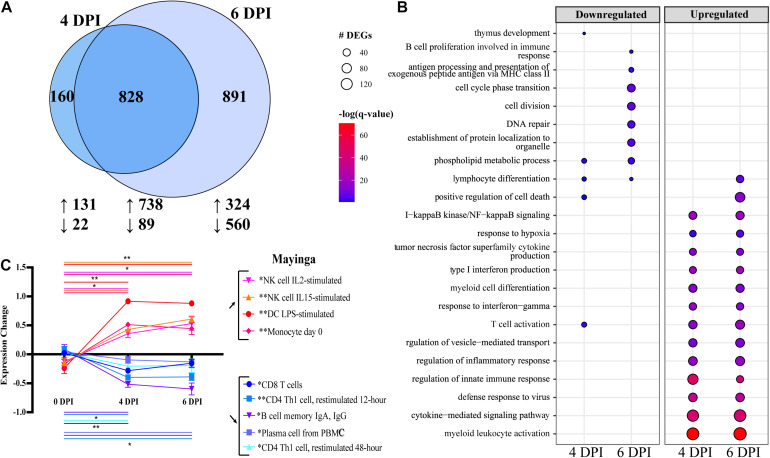
Differential gene expression analysis with EdgeR following EBOV-Mayinga infection in rhesus macaques shows robust immune activation and dysregulation. **(A)** Venn diagram of number of differentially expressed genes (DEGs) (FDR ≤ 0.05, fold change ≥ 1 or 1, rpkm 5; see section “Materials and Methods”) detected at 4 and 6 DPI following EBOV-Mayinga infection. The number of upregulated and downregulated DEGs is indicate with upward- and downward-pointing arrows, respectively. **(B)** Bubbleplot summarizing functional enrichment of DEGs up- and downregulated 4 and 6 DPI. Color intensity of each bubble represents the negative log of the FDR-adjusted *p*-value [–log(*q*-value)], and the relative size of each bubble represents the number of DEGs belonging to the specified gene ontology (GO) term. **(C)** Digital cell quantification analysis using DMAP and IRIS datasets. Lines are color-coded to match both the subset listed in the legend. Significance lines of which both ends denote the two time points of the two-way multiple comparisons significance testing. Asterisks in legend cell population denote overall significance (0–4 DPI, 4–6 DPI, and 0–6 DPI) through the mixed effects model test. *p < 0.05, **p < 0.01, ***p < 0.001.

No studies to date have examined the transcriptional response to EBOV-Mayinga, the first EBOV isolate identified in 1976 (CFR of ∼90%). Therefore, we leveraged access to historical WB RNA samples from our previous study to profile host responses at 0, 4, and 6 DPI (Marzi et al., 2018). Significant transcriptional changes were identified with a total of 985 and 1,721 DEGs detected 4 and 6 DPI, respectively, with a substantial overlap between the two time points (Figure 1A). Functional enrichment of these DEGs using Metascape showed that DEGs downregulated at 4 and 6 DPI enriched to GO terms related to adaptive immunity (Figure 1B) such as “thymus development” (e.g., *AGER*, *IL7R*, and *ZAP70*) and “T cell activation” (e.g., *AKT1*, *BTLA*, and *TCF7*) (Figure 1B and Supplementary Figure 1A) (Zhou et al., 2019). DEGs downregulated at 6 DPI enriched to “antigen processing and presentation of exogenous peptide antigen *via* MHC class II” (e.g., *HLA-DMA*, *-DMB*, and *-DOB*) and “B cell proliferation” (e.g., *CD19*, *CD180*, and *PLCL2*) (Figure 1B and Supplementary Figure 1A). In addition, DEGs downregulated at 6 DPI played a role in “DNA repair” (e.g., *ATM*, *DNA2*, and *POLE2*) and “cell cycle phase transition” (e.g., *BUB1B*, *CDC27*, and *CKAP5*) (Figure 1B).

In contrast to downregulated DEGs, upregulated DEGs detected at 4 and 6 DPI enriched predominantly to GO terms related to innate immunity and inflammation, notably “myeloid cell differentiation” (e.g., *BATF2*, *MAFB*, *NFE2 < TLR2*, *TLR3*, and *RELB*) (Figure 1B and Supplementary Figure 1B). DEGs enriching to “type I interferon (IFN) production” consisted primarily of IFN-stimulated genes (ISG; e.g., *IFI16*, *IRF7*, *ISG15*, and *STAT1*), as well as genes involved in detection and response to pathogenic nucleic acid (e.g., *DHX58*, *DDX58*, *IRF7*, and *TBK1)* (Supplementary Figure 1C). Finally, some upregulated DEGs at 4 and 6 DPI also enriched to “T cell activation” (e.g., *CD274*, *IL2RA*, *LFNG*, and *TCIRG1*) (Supplementary Figure 1D).

We next sought to correlate these transcriptional changes with alterations in immune cell frequencies and activation status. Since flow cytometry was not conducted in the earlier study and no PBMC were cryopreserved, we performed DCQ to predict changes in immune cell population frequencies based on our transcriptional data (Figure 1C). Using the IRIS immune cell database, transcriptional findings predicted sharp decreases in the frequencies of T and B cells (particularly antibody-secreting B cells), while the number of myeloid cells and natural killer (NK) cell populations was predicted to increase with disease progression (Figure 1C).

**FIGURE 2:**
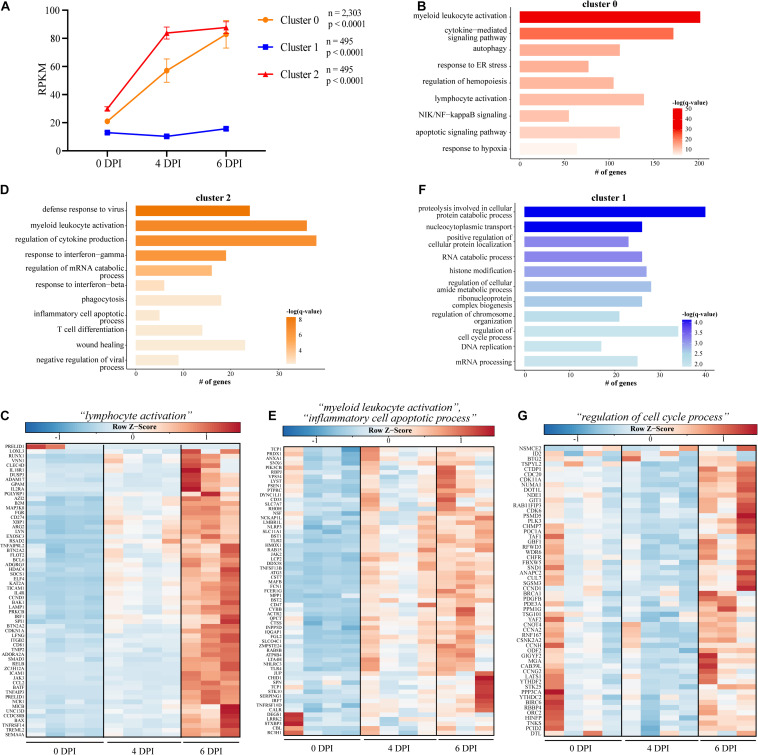
Short Time Series Expression Miner (STEM) analysis of gene expression data following EBOV-Mayinga infection. **(A)** Gene expression clusters identified by STEM with accompany number (*n*) of genes and *p*-value (*p*). GO term bar graphs representing functional enrichment of the genes from **(B)** cluster 0, **(D)** cluster 2, and **(F)** cluster 1. Horizontal bars represent the number of genes mapping to each GO term, while color intensity represents the –log(*q*-value) value of the corresponding GO term. Gene expression heatmaps depicting genes from GO term **(C)** “lymphocyte activation” in cluster 0, **(E)** “myeloid leukocyte activation” and “inflammatory cell apoptotic process” in cluster 2, and **(G)** “regulation of cell cycle process” from cluster 1. Each column represents one animal. Red represents upregulation; blue, downregulation. Range of colors is based on scaled and centered rpkm values of the represented DEGs.

Given that bivariate analysis of the transcriptional changes at each DPI relative to baseline does not consider the dynamic, longitudinal patterns of gene expression, we next used Short Time-series Expression Miner (STEM) to identify clusters of genes, the expression of which changes in a similar manner over time (Figure 2) (Ernst and Bar-Joseph, 2006). We identified three clusters with genes in clusters 0 and 2 significantly upregulated throughout infection, while expression of genes in cluster 1 was slightly decreased 4 DPI before returning to baseline levels of expression at 6 DPI (cluster 1) (Figure 2A). Genes in cluster 0 (*n* = 2,303) enriched to GO terms related to the innate inflammatory response such as “myeloid leukocyte activation” (e.g., *CD14*, *CD53*, and *CD55*), “cytokine-mediated signaling pathway” (e.g., *IFNAR2*, *IL15RA*, *JUNB*, and *LTB*), “NIK/NF-kappaB signaling” (e.g., *NFKB2*, *RELA*, *TRAF2*, and *TLR9*), and “apoptotic signaling pathway” (e.g., *CASP1*, *CASP4*, and *FAS*) (Figure 2B). Genes also enriched to GO term “lymphocyte activation” and were related to both cellular (e.g., *CD274*, *JAK3*, *TNFRSF14*, and *TREML2*) and humoral (e.g., *BCL6*, *LYN*, and *PRKCB*) immunity (Figures 2B,C). Cluster 2 (*n* = 495) was comprised of genes that enriched to GO terms indicative of heightened inflammatory state such as “positive regulation of tumor necrosis factor production” (e.g., *JAK2* and *TNFRSF1A*), “myeloid leukocyte activation” (e.g., *FCER1G*, *NLRP3*, and *TLR4*) (Figure 2D), and “defense response to virus” (e.g., *BST2*, *AIM2*, and *HERC5*) (Figure 2E). Lastly, the downregulated genes of cluster 1 (*n* = 495) were involved in metabolic and cellular processes such as “regulation of cell cycle process” (e.g., *ANAPC2*, *CCND1*, and *CDC20*) and “RNA catabolic process” (e.g., *AGO1*, *LARP1*, and *PYM1*) (Figures 2F,G).

### Transcriptional Responses to Late EBOV-Makona Isolates Indicate Attenuated Inflammation and Lymphopenia

**FIGURE 3:**
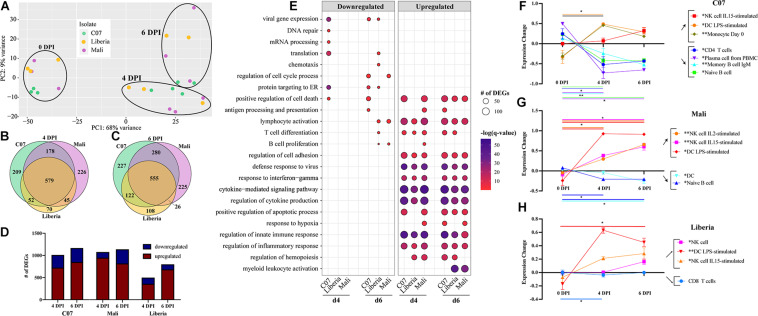
Host response comparison between early and late isolates of EBOV-Makona. **(A)** Principal component analysis of normalized transcript counts at 0, 4, and 6 DPI for EBOV-Makona isolates. Venn diagrams of number of differentially expressed genes (DEGs) (FDR 0.05, fold change ≥ 1 or 1, RPKM 5) identified through EdgeR following infection with either EBOV-Makona Guinea C07, Liberia, or Mali at **(B)** 4 DPI and **(C)** 6 DPI. **(D)** Bar graph of DEGs detected at 4 and 6 DPI for each isolate. **(E)** Bubbleplot presenting functional enrichment of upregulated and downregulated DEGs detected in each isolate at 4 and 6 DPI. The size of the bubble represents the number of DEGs, while the color represents the –log(*q*-value). ImmQuant analysis using DMAP and IRIS datasets for **(F)** C07, **(G)** Mali, and **(H)** Liberia. Lines are color-coded on the graph to match both the subset listed in the legend and the significance lines, of which both ends denote the two time points of the two-way multiple comparisons significance testing. A “*” over a legend denotes overall significance through the mixed effects model test. *p < 0.05, **p < 0.01, ***p < 0.001.

Next, we examined transcriptional changes following challenge with early (Guinea C07) and late (Liberia and Mali) EBOV-Makona isolates (Figure 3). Disease progression and clinical data for these animals were reported in our previous study and indicate uniform lethal infection, albeit slightly delayed following infection with late isolates in rhesus macaques (Marzi et al., 2018). Comparative bivariate transcriptional analysis revealed striking similarities in the overall magnitude and character of the transcriptional response to early and late isolates (Figures 3A–C). The majority of DEGs detected 4 DPI and 6 DPI following challenge with each isolate overlapped, with a greater number of DEGs detected 6 DPI compared to 4 DPI, and most DEGs were upregulated (Figures 3B–D). Liberia exhibited a particularly lower number of DEGs (∼500) at 4 DPI compared to other isolates (>1,000 DEGs) (Figure 3B). These downregulated DEGs enriched to GO terms related to gene expression, including “translation,” “mRNA processing,” and “protein targeting to ER” (Figure 3E). On the other hand, DEGs downregulated by 6 DPI played a role in cell cycle (C07, Mali), adaptive immunity (C07, Liberia, Mali), translation (Liberia), and apoptosis (C07) (Figure 3E). Functional enrichment of upregulated DEGs at 4 and 6 DPI is consistent with a heightened inflammatory response with over-representation of GO terms related to EVD pathology (e.g., “defense response to virus,” “positive regulation of cell death,” and “regulation of cytokine production”) (Figure 3E).

Given that DEGs detected 4 and 6 DPI following infection with all three isolates enriched to similar GO terms, we next examined the overlap between those sets of DEGs (Supplementary Figure 2). DEGs that enriched to GO term “positive regulation of cell death” were upregulated throughout infection and showed significant overlap (Supplementary Figure 2A). These genes played a role in apoptosis (e.g., *CASP1*, *FAS*, and *TNFRSF1A*) and inflammation (e.g., *IL6*, *NLRP3*, *TLR4*, and *TNF*) (Supplementary Figure 2A). Similarly, many upregulated DEGs enriching to “defense response to virus” were shared between the isolates and encoded nucleic acid sensors (e.g., *DDX58*, *DDX60*, and *OAS1*), components of the IFN signaling pathway (e.g., *STAT1*, *STAT2*, and *TBK1*), and ISGs (e.g., *AIM2*, *IFI16*, and *ISG15*) (Supplementary Figure 2B). Shared DEGS that enriched to “regulation of cytokine production” included a mixture of genes important for inflammation (e.g., *IL18R1* and *MYD88*), chemotaxis (e.g., *FLOT1* and *ROCK2*), and pathogen recognition (e.g., *CLEC7A*, *CLEC4A*, and *TLR2*) (Supplementary Figure 2C). DEGs upregulated at 4–6 DPI in all isolates and enriching to “lymphocyte activation” reflected both B and T cell-mediated immunity (e.g., *ARG2*, *BCL6*, *LYN*, and *STAT3*) (Supplementary Figure 2D). Similarly, DEGs belonging only to C07 and Liberia for this term also associated with lymphocyte-mediated immunity (e.g., *CD81*, *IL6*, and *LCP1*) as well as T cell regulation (e.g., *IDO1*, *PDCD1LG2*, and *TCIRG1*) (Supplementary Figure 2D).

Complete blood count analysis showed hallmarks of EVD in all four cohorts of animals including lymphopenia, neutrophilia, and thrombocytopenia (Supplementary Figure 3 and Supplementary Table 1). Furthermore, significant declines in platelets were noted as early as 2 DPI but only in Mayinga-infected animals (Supplementary Figure 3 and Supplementary Table 1). Declines in lymphocyte numbers were noted only in Mayinga and C07 infections at 4 DPI (Supplementary Figure 3 and Supplementary Table 1). However, lymphopenia was most pronounced in Mayinga-infected animals (Figure 3B), whereas neutrophilia was not evident in animals infected with the late EBOV-Makona variant Mali (Figure 3C). To gain a deeper understanding of the changes in immune cell frequencies following infection with EBOV-Makona isolates, we performed DCQ. Frequencies of activated NK and DC subsets were predicted to increase over the course of infection for all three isolates (Figures 3F–H). Higher frequencies of monocytes were only predicted following C07 infection (Figure 3F). Significant lymphopenia was predicted to occur following infection with C07 wherein levels of CD4 T cells and several B cell subsets decreased with infection (Figure 3F). In contrast, significant but modest changes in naïve B cells or CD8 T cells were predicted to occur following infection with Mali and Liberia variants, respectively (Figures 3G–H).

We next used STEM to identify groups of genes with similar patterns of longitudinal gene expression (Supplementary Figure 4). In all isolates, we detected a cluster (cluster 0) of genes whose expression progressively increased over the course of infection (Supplementary Figures 4A–C). Genes in cluster 0 from all three isolates enriched to similar GO terms involved in both innate (e.g., “myeloid leukocyte activation” and “regulation of innate immune response”) and adaptive (e.g., “lymphocyte activation” and “T cell differentiation”) immunity (Supplementary Figure 4D). A second cluster (cluster 1) featured genes whose expression levels were slightly downregulated at 4 DPI before returning to near baseline by 6 DPI and enriched to “translation” and “DNA repair” (Supplementary Figure 4E). However, a unique cluster (cluster 2) was identified following infection with the EBOV-Makona Guinea C07 isolate, which consisted of genes robustly upregulated at 4 and 6 DPI. Genes in this cluster played roles in innate immunity (e.g., “myeloid leukocyte activation”), adaptive immunity (e.g., “T cell receptor signaling pathway”), antiviral defense (e.g., “cellular response to type I IFN”), and EVD pathology (e.g., “blood coagulation”) (Supplementary Figure 4F).

### Ebola Virus-Makona and EBOV-Mayinga Induce an Overlapping Core of Antiviral, Inflammatory, and Apoptotic Genes

**FIGURE 4:**
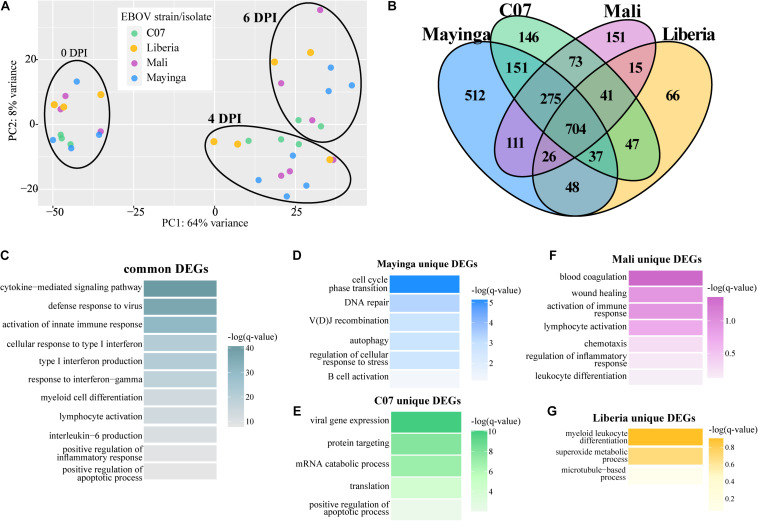
Ebola virus-Makona and EBOV-Mayinga isolates share a core of DEGs related to EVD pathology. **(A)** Principal component analysis of normalized transcript counts at 0, 4, and 6 DPI for EBOV-Makona isolates and EBOV-Mayinga. **(B)** Four-way Venn diagram depicting overlaps between all up- and downregulated DEGs identified 4 and 6 DPI for all isolates. Functional enrichments for DEGs **(C)** common to all isolates or unique to **(D)** EBOV-Mayinga, **(E)** Guinea C07, **(F)** Mali, and **(G)** Liberia. Color indicates the –log(*q*-value) of each GO term.

We next compared transcriptional changes among animals infected with EBOV-Mayinga or EBOV-Makona isolates (Figure 4). PCA showed a significant overlap between all four infections at each DPI (Figure 4A). We identified a shared core of 704 genes that were involved in antiviral immunity (e.g., *IFIH1*, *IRF7*, *OAS1*, *ISG15*, and *STAT1* – GO terms “defense response to virus,” “cellular response to type I IFN”), inflammation (e.g., *NOD2*, *TICAM1*, and *TLR4* – GO terms “activation of innate immune response,” “myeloid cell differentiation”), cytokine signaling (e.g., *CCL4*, *INPP5D*, and *IRAK2* – GO term “cytokine-mediated signaling pathway”), and immune cell activation (e.g., *LYN*, *MAFB*, *PRKCB*, and *VAV1* – GO terms “lymphocyte activation”) (Figures 4B,C).

Additionally, there were DEGs unique to each infection. The largest group of unique DEGs was detected following EBOV-Mayinga infection. Those unique DEGs were associated with B cell-mediated immunity (e.g., *ATM*, *CD27*, *PRKDC*, and *TCF3*), stress responses (e.g., *BAX*, *EYA3*, and *NUP155*), and DNA repair (e.g., *COPS2*, *LIG3*, and *PYHIN1*) (Figure 4D). DEGs detected only following infection with the early EBOV-Makona Guinea C07 isolate played a role in nucleic acid metabolism (e.g., GO term “viral gene expression” and “translation”) and catabolic stress responses (e.g., GO term “positive regulation of apoptotic process”) (Figure 4E). Many DEGs unique to late EBOV-Makona Mali infection enriched to GO terms related to EVD associated processes, notably “blood coagulation,” “regulation of inflammatory response,” and “wound healing” (Figure 4F). The few DEGs unique to late EBOV-Makona Liberia infection played a role in myeloid cell differentiation (e.g., *HCLS1* and *RARA*) and host metabolic processes (e.g., *CCS* and *PN3*) (Figure 4G).

**FIGURE 5:**
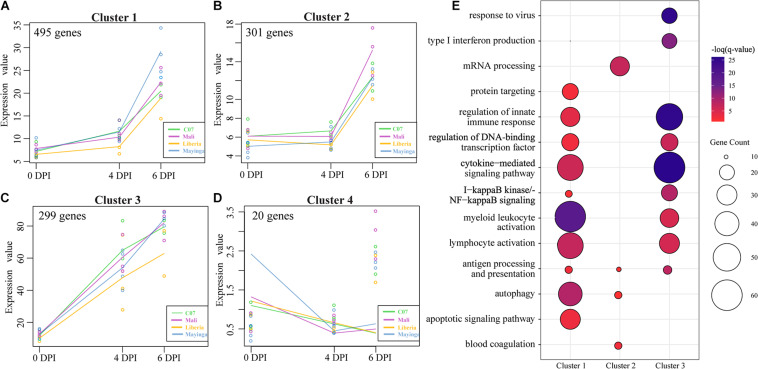
MaSigPro, two-way forward regression analysis of EBOV-Makona and EBOV-Mayinga isolates. **(A–D)** Gene clusters 1–4 identified by MaSigPro. **(E)** Bubbleplot depicting functional enrichment of genes belonging to clusters 1, 2, and 3. The size of the bubble represents the number of DEGs, while the color represents the –log(*q*-value).

To identify clusters of genes with similar patterns of gene expression across all EBOV-Makona and EBOV-Mayinga isolates, we applied a two-way forward regression model using MaSigPro (Figure 5) (Conesa et al., 2006). We retained only those genes that were considered significant in at least 16 comparisons and then clustered them based on temporal expression patterns, which results in four significant clusters (Figures 5A–D). Genes in clusters 1 and 2 exhibited modest upregulation 4 DPI followed by a sharp increase 6 DPI. These 495 genes played a role in the activation and regulation of innate (e.g., “regulation of innate immune response”) and adaptive (e.g., “lymphocyte activation”) immune responses (Figures 5A,E). Notable genes within this cluster are involved in leukocyte–leukocyte interactions (e.g., *ICAM1* and *ITGB2*), myeloid cell signaling (e.g., *CD14*, *RELA*, *NFKB1*, and *TRAF3*), as well as lymphocyte activation (e.g., *BATF*, *LAT2*, and *PRKCB*) (Supplementary Figure 5A). The 301 genes in cluster 2 mainly played a role in translation (“mRNA processing”), antigen presentation (“autophagy” and “antigen processing and presentation”), and coagulopathy (“blood coagulation”; e.g., *A2M*, *P2RX1*, *PRKAR2B*, and *SELP*) (Figures 5B,E and Supplementary Figure 5B). Expression of genes in cluster 3 progressively increased from 0 to 6 DPI and enriched to similar GO processes described for clusters 1 and 2 in addition to “type I IFN production”/“response to virus” (e.g., *DDX58*, *IFIH1*, *IRF1*, *STAT1/3*, and *ISG15*) (Supplementary Figure 5C). Cluster 4 consisted of 20 genes associated with metabolism that were mostly downregulated over the course of infection (Figure 5D).

**FIGURE 6:**
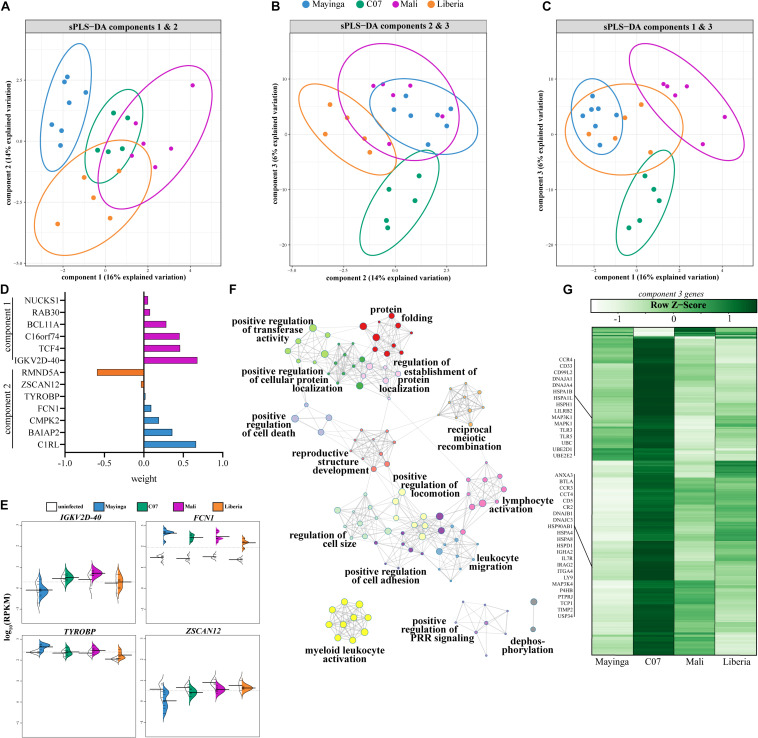
Genes regulating immune and stress responses are sufficient to distinguish EBOV infections. **(A–C)** Plots depicting the three components contributing the most to the variation amongst infected animals at 4 and 6 DPI as determined by sparse partial least squares discrimination analysis (sPLS-DA). **(D)** Weight loadings of genes belonging to components 1 and 2 as identified in panels **(A–C)**. **(E)** Beanplots illustrating expression (log10RPKM) of genes from panel D. **(F)** Gene ontology (GO) network depicting functional enrichment of 290 genes of component 3. Clusters of closely related GO terms are labeled with the most significant GO term. Node size represents the number of genes associated with the GO term. Gray lines represent shared interactions between GO terms, with density and number indicating the strengths of connections between closely related GO terms. **(G)** Heatmap representing the 290 genes of component 3. Each column represents the average rpkm values of the indicated group across 4 and 6 DPI. Range of colors is based on scaled and centered rpkm values of the represented DEGs.

To identify gene signatures that can differentiate among the groups, we next applied a sparse partial least-squares discrimination analysis (sPLS-DA) (Figure 6). Contrary to PCA, sPLS-DA permits identification of the minimum number of genes responsible for driving a given component of variation while preserving maximum covariance among defined groups. We identified three components that contributed to the majority of variation among EBOV strains (Figures 6A–C). The six genes that explained component 1 (16% variation) included immunoglobulin light chain *IGKV2D-40*, intracellular trafficking protein *RAB30*, and transcription factor *TCF4* that regulates lymphoid and plasmacytoid DC development (Figures 6A–D). These six genes were sufficient to separate EBOV-Mayinga and EBOV-Makona isolates, but this separation was heavily influenced by Mali infection (Figures 6D,E). The six genes that explained component 2 (14% variation) separated Liberia from EBOV-Mayinga infections (Figures 6A–D). These six genes played a role in immune signaling (e.g., *FCN1* and *TYROBP*) and ubiquitin-protein ligase complex component (*RMND5A*) and were upregulated with EBOV-Mayinga and Liberia infection, respectively (Figure 6E). Nearly 300 genes belonging to component 3 (6% variation) distinguished C07 from all other EBOV infections (Figures 6B,C). These genes played a role in host defense as well as cellular homeostasis, as indicated by enrichment to GO terms “myeloid leukocyte activation” (e.g., *TLR3*, *TLR5*, *CR2*, and *CCR3*), “lymphocyte activation” (e.g., *IGHA2*, *IL7R*, *ITGA4*, and *LILRB2*), “protein folding” (e.g., *UBC* and *UBE2D1*), and “positive regulation of cellular protein localization” (e.g., *MAPK1* and *TIMP2*) (Figures 6F,G). This included a large number of chaperones and ubiquitin-conjugating enzymes (e.g., *DNAJA1*, *HSPA1B*, and *UBE2D1*) and genes modulating lymphocyte-mediated immunity (e.g., *IGHA2*, *IL7R*, *ITGA4*, and *LILRB2*) that were most prominently expressed in C07 infection (Figure 6G).

## Discussion

Recent epidemiological studies suggest that different EBOV strains are associated with significant differences in CFRs despite sharing over 97% genetic similarity (Bell et al., 2015; Marzi et al., 2015, 2018; Wong et al., 2016; Menicucci et al., 2017; Versteeg et al., 2017, 2019; Madelain et al., 2018; McMullan et al., 2019). Specifically, historical strains EBOV-Mayinga and EBOV-Kikwit incur CFRs near 90%, while the EBOV-Makona strain from the 2013–2016 West Africa epidemic incurred a notably lower CFR of ∼40% (Centers for Disease Control and Prevention, 1995a, b; Muyembe-Tamfum et al., 1999; Kuhn et al., 2013; Zhang and Scheuermann, 2014; Kaner and Schaack, 2016; Shoman et al., 2017). Over the course of the 2013–2016 West Africa epidemic, several isolates of EBOV-Makona emerged late (Mali, Liberia) in 2014, which contained mutations in key genes (e.g., GP, NP, and the RNA dependent RNA polymerase) that were associated with changes in replication kinetics *in vitro* and were believed to facilitate widespread dissemination of the virus. However, *in vivo* studies in NHP did not show increased virulence of these variants, but rather a modest delay to euthanasia in a uniformly lethal challenge model (Marzi et al., 2018). Additionally, the CFR late in the epidemic was not higher than that observed earlier in the epidemic (Garske et al., 2017; Forna et al., 2020). It is likely that the lower CFR observed during the West Africa Epidemic is due to improved public health intervention strategies and mobilization. Indeed, CFRs for recent EBOV outbreaks in the DRC (2017–2020) are also lower than historical CFRs, ranging from ∼40 to 66% across 3,662 reported cases (Centers for Disease Control and Prevention, 2021b). However, the potential impact of these mutations on host immunity and defense has yet to be defined. These studies would provide key insight into the interaction between viral genetic evolution on host pathogenesis.

Therefore, in this study, we conducted the first comparative analysis of host molecular responses to infection with EBOV-Mayinga and EBOV-Mayinga infections using an NHP model (Marzi et al., 2018). We leveraged access to historical RNA samples and used RNA-Sequencing to compare transcriptional responses in the WB of rhesus macaques infected with a lethal dose of either EBOV-Mayinga or early (C07) or late (Mali, Liberia) EBOV-Makona isolates. Bivariate, longitudinal, regression, and discrimination analysis strategies allowed us to identify key to comprehensively identify differences and similarities among host responses.

Our analysis of the transcriptional response to EBOV-Mayinga revealed transcriptional changes reflecting canonical characteristics of EVD such as progressive upregulation of genes associated with inflammation, apoptosis, and antiviral defense, while genes associated with adaptive immunity were primarily suppressed. These observations are in line with the severe lymphopenia, myelopoiesis, and the cytokine storm that constitute the hallmarks of EVD (Geisbert et al., 2003a; Rivera and Messaoudi, 2016; Jacob et al., 2020). These results support previous analyses of host transcriptional responses to EBOV-Kikwit in cynomolgus macaques and fatal EBOV infection in humans (Barrenas et al., 2015; Eisfeld et al., 2017; Liu et al., 2017; Menicucci et al., 2017; Versteeg et al., 2017; Kotliar et al., 2020).

As described for EBOV-Mayinga infection, transcriptomes of NHPs infected with early or late EBOV-Makona isolates indicated progressive upregulation of genes related to innate immune activation, inflammation, and innate antiviral immunity. A core of ISGs was longitudinally upregulated following infection with all isolates and confirmed by regression analysis. This was paralleled by predicted increases in the frequencies of activated DCs and monocytes by DCQ analysis. Although our analysis could not resolve plasmacytoid (p)DCs, the major source of type I IFN, previous analysis showed that circulating IFN-alpha levels in the animals studied here were significantly elevated at 6 DPI for all EBOV-infected NHPs (Marzi et al., 2018). A sustained antiviral IFN and cytokine response has been shown to be detrimental to the host by suppressing adaptive immunity in acute and chronic viral infections and inducing bystander death of T and B cells, which is not seen in the bat reservoir of EBOV (Geisbert et al., 2000, 2003a; Bradfute et al., 2008; Stacey et al., 2009; O’Brien et al., 2011; Davidson et al., 2015). Recent transcriptional profiling of WB RNA samples from EBOV-infected human patients also identified a strong inflammatory ISG signature as a predictor of fatal outcome (Villinger et al., 1999; Baize et al., 2002; Wauquier et al., 2010; McElroy et al., 2014; Liu et al., 2017). Interestingly, the expression of *TCF4*, a transcription factor highly expressed in pDCs, was sufficient to distinguish EBOV-Makona (especially late isolate Mali) from EBOV-Mayinga infections (Forrest et al., 2014). This observation suggests that activation of pDCs may be associated with reduced mortality. The aberrant IFN production associated with fatal EBOV outcome could be primarily mediated by non-pDC cells that are infected by EBOB such as endothelial cells. All EBOV-Makona isolates also featured increases in activated NK cells and inflammatory DCs, which may contribute to EVD pathology (Warfield et al., 2004; Mohamadzadeh et al., 2006; Cimini et al., 2017; Menicucci et al., 2017; Fausther-Bovendo et al., 2019; Kotliar et al., 2020).

Our discrimination analysis did not identify inflammatory genes as main drivers of transcriptional distinction among EBOV infections, suggesting that the cytokine storm is a conserved outcome. Rather, genes involved in host defense, such as *FCN1* and *TYROBP* expressed in EBOV-Mayinga infection, and numerous protein chaperones highly upregulated in EBOV-Makona C07 infection, were sufficient to separate infections. Protein folding in response to cellular stress can be either beneficial or harmful during viral infection since increases in the levels of protein chaperones may facilitate the folding of viral proteins or the expression of host antiviral defense proteins (Maruri-Avidal et al., 2008; Khachatoorian et al., 2016; Pujhari et al., 2019; Paladino et al., 2020; Wan et al., 2020). This finding complements our longitudinal (STEM) and bivariate analyses that identified sets of genes uniquely expressed during EBOV-Makona C07 infection that associate with protein localization and cellular stress. This distinction provides a potential explanation for the delayed disease progression following EBOV-Makona C07 infection relative to EBOV-Mayinga.

Interestingly, genes related to type I IFN response, apoptosis, cell death, and inflammatory signaling pathways were less upregulated initially following infection with the EBOV-Makona Liberia isolate. These differences were in line with the lack of neutrophilia and reduced levels of serum IFN-alpha in this group of animals. A delay in these transcriptional changes may contribute to minor differences in disease pathology, such as prolonged time to death in some NHPs, and the lack of lymphopenia predicted using DCQ (Marzi et al., 2018). Additionally, genes related to hemopoiesis were upregulated early during infection, which may result in the reduced severity of lymphopenia observed in this group. Although macaques infected with the EBOV-Makona Liberia isolate ultimately succumb to disease, the delay in clinical disease progression may provide a larger window for clinical intervention (Marzi et al., 2018). Furthermore, subversion of the host response may also permit viral persistence, as seen by the reemergence of an EBOV isolate closely related to EBOV-Makona Liberia in a survivor from the 2013–2016 outbreak (Centers for Disease Control and Prevention, 2021a).

Genes associated with adaptive immunity were largely downregulated in all animals, albeit more pronounced following EBOV-Mayinga, followed by EBOV-Makona Guinea C07 infection compared to other EBOV-Makona isolates. Furthermore, we detected significant declines in CD4 Th1 and CD8 populations in only EBOV-Mayinga infection that paralleled robust apoptotic gene expression. The lymphopenia predicted based on gene expression agrees with our previous flow cytometry analysis of PBMC from EBOV-Makona infected cynomolgus macaques (Versteeg et al., 2017). Interestingly, discrimination analysis identified a number of T cell-related genes (e.g., *IL7R*, *LY9*, and *TCP1*) that were uniquely upregulated with Makona-C07 infection, which may reflect aberrant T cell activation associated with fatal outcomes in EVD patients (Baize et al., 1999; Agrati et al., 2016). The expression of these genes could potentially explain the reduced severity in lymphopenia and CFR compare to Mayinga infection. Nevertheless, minor dysregulation in T cell responses can have significant ramifications for disease progression. Dysregulated T cell and cytotoxic responses are associated with a poor EVD prognosis, which may be driven by excessive production of type I IFN interferon (Agrati et al., 2016; Ruibal et al., 2016; Speranza et al., 2018).

Humoral immunity is also critical for recovery from and long-term protection against EVD (Thom et al., 2020). Monoclonal antibodies targeting the EBOV GP, as well as antibodies formed following vaccination with the FDA-approved EBOV vaccine, are also known to be protective in both humans and NHPs (Maruyama et al., 1999; Corti et al., 2016; Holtsberg et al., 2016; Misasi et al., 2016; Gaudinski et al., 2019). EBOV-Makona and EBOV-Mayinga isolates induced upregulation of genes involved in humoral immunity, although these gene sets were largely distinct. The loss of lymphocytes and predicted decline in B cell populations in was most pronounced in EBOV-Mayinga, followed by EBOV-Makona Guinea C07 and Mali, but not EBOV-Makona Liberia.

The major caveat of our study is that lethal doses of EBOV were administered to each animal, resulting in rapid disease progression not seen in typical human infections. This prevents resolution of finer differences in disease progression and restricts longitudinal analysis, which should be addressed in future studies by using nonlethal doses. Another caveat is that mutations identified during a human outbreak may not elicit the same response from rhesus macaques’ immune system. Nevertheless, this is the first study to compare molecular host responses using transcriptomics within the WB of animals infected with the historical EBOV-Mayinga isolate or the EBOV-Makona isolates that arose during the recent 2013–2016 West Africa epidemic. Our data provide critical insight into the impact of genetic variation among and between strains of EBOV on the molecular host response to infection in NHPs. Our transcriptional analyses indicate similar patterns of gene expression related to immune activation, inflammation and cell death, and induction of a sustained core of innate antiviral genes following infection with all EBOV isolates. However, late EBOV-Makona isolates Mali and Liberia were associated with less severe lymphopenia and smaller transcriptional responses than earlier EBOV-Makona isolates that are not reflected as disease attenuation at the clinical level. Later EBOV-Makona isolates also lacked the sharp upregulation of pathology-associated genes/cytokine storm seen in early EBOV-Makona isolate infections. However, a small subset of genes (∼300) that play a role in innate and adaptive immunity was sufficient to distinguish one infection from another, suggesting potential differences in immune responses. While transcriptional differences exist, all infections result in lethal outcomes. Therefore, viral genetic variation is associated with distinct molecular EVD pathogenesis, but not outcome implying that this cannot be a major factor influencing the 2013–2016 epidemic transmission rate or reduced CFR.

## Data Availability Statement

Sequencing data for rhesus macaques is available at BioProject PRJNA718880.

## Ethics Statement

The animal study was reviewed and approved by the Rocky Mountain Laboratory Animal Care and Use Committee.

## Author Contributions

KM, AP, IM, and AM designed the experiments. KM, AP, and AM conducted the experiments. KM, AP, and IM analyzed the data and wrote the manuscript. All authors approved the manuscript.

## Conflict of Interest

The authors declare that the research was conducted in the absence of any commercial or financial relationships that could be construed as a potential conflict of interest.

## Publisher’s Note

All claims expressed in this article are solely those of the authors and do not necessarily represent those of their affiliated organizations, or those of the publisher, the editors and the reviewers. Any product that may be evaluated in this article, or claim that may be made by its manufacturer, is not guaranteed or endorsed by the publisher.

## Footnotes

1
https://royalsociety.org/policy/publications/2006/weatherall-report/

## Supplementary Material

Supplementary Figure 1 Analysis of EBOV-Mayinga infection DEGs enriching to key GO terms. **(A)** Heatmap of downregulated genes enriching to “Thymus development,” “B cell proliferation involved in immune response,” “Antigen processing and presentation of exogenous peptide antigen via MHC class II,” “Lymphocyte differentiation,” and “T cell activation.” Membership of genes to gene ontology (GO) terms is denoted by matching colored marker. Heatmaps of 4 DPI unique, 6 DPI unique, or shared upregulated genes enriching to **(B)** “Myeloid cell differentiation,” **(C)** “T cell activation,” and **(D)** “Type I interferon production.” Each column represents one animal. Red presents upregulated; blue represents downregulated. Range of colors is based on scaled and centered rpkm values of the represented DEGs.

Supplementary Figure 2 Comparison of upregulated DEGs enriching to GO terms shared by early and late EBOV-Makona isolates. Heatmaps of shared upregulated DEGs enriching to GO terms common to all isolates: **(A)** “positive regulation of cell death,” **(B)** “defense response to virus,” **(C)** “regulation of cytokine production,” and **(D)** “lymphocyte activation.” Accompanying Venn diagrams depict the DEGs enriching to each isolate per given the given GO term. Only DEGs shared by either all isolates [e.g., panel **(A)**] or by Guinea C07 and Mali [e.g., panel **(D)**] are depicted and are indicated in red in the Venn diagram. Each column represents one animal. Red presents upregulated; blue represents downregulated. Range of colors is based on scaled and centered rpkm values of the represented DEGs.

Supplementary Figure 3 Ebola virus infection results in lymphopenia, neutrophilia, and thrombocytopenia. **(A)** Total number of white blood cells (thousand/μl), **(B)** lymphocytes (% total cells), **(C)** neutrophils (%) total cells, and **(D)** platelet count (thousand/μl) for EBOV-Mayinga and EBOV-Makona isolates from d0 to d6. Significant differences between isolates at a given time point were determined with one-way ANOVA with multiple comparisons. ^∗^Guinea C07, #Mali, $Liberia. *p*-value < 0.05^∗^, *p*-value < 0.01^∗∗^, *p*-value < 0.001^∗∗∗^, and *p*-value < 0.0001^****^.

Supplementary Figure 4 STEM analysis of EBOV-Makona isolates. Gene expression graphs depicting the STEM clusters identified for EBOV-Makona **(A)** Guinea C07, **(B)** Mali, and **(C)** Liberia. GO term enrichment heatmaps for **(D)** cluster 0 and **(E)** cluster 1. Color intensity represents the statistical significance [shown as the −log(*q*-value)] with the range of colors based on GO terms with the lowest and highest significance statistic for the entire set of GO terms per cluster. The number of genes enriching to each GO term per column is represented in each box; white boxes indicate no statistical significance. **(F)** GO term bar graphs representing enrichment of genes in cluster 3. Horizontal bars represent the number of genes mapping to each GO term, while color intensity represents the −log(*q*-value) value of the corresponding GO term. Panels **(D–F)** follow the color key in plots A–C: cluster 0, red; cluster 1, blue; and cluster 2, orange.

Supplementary Figure 5 Analysis of genes identified by MaSigPro, two-way forward regression analysis for EBOV-Makona and EBOV-Mayinga isolates. Heatmaps of genes identified by MaSigPro for **(A)** cluster 1, **(B)** cluster 2, **(C)** cluster 3, and **(D)** cluster 4. The latter was split into two sub-clusters: cluster 4A containing genes that were upregulated only in response to Liberia isolate and cluster 4B containing genes downregulated by all isolates. Select genes are indicated. Each column represents the median RPKM of all animals at a given day post infection. Red presents upregulated; blue represents downregulated. Range of colors is based on scaled and centered rpkm values of the represented DEGs.

Supplementary Table 1 Statistical analysis (adjusted *p*-values) of longitudinal changes in cell and platelet populations. Adjusted *p*-values for data in Supplementary Figure 3. Two-way ANOVA with Geisser-Greenhouse correction to account for unequal variability of differences. Significant values highlighted in red. DPI, days post infection.
