# Supplementary figures and images for: Transcriptional Analysis of Infection With Early or Late Isolates From the 2013–2016 West Africa Ebola Virus Epidemic Does Not Suggest Attenuated Pathogenicity as a Result of Genetic Variation
Source: Front Microbiol. 2021 Aug 13;12:714817. doi: 10.3389/fmicb.2021.714817 (PMC8415004; doi:10.3389/fmicb.2021.714817)

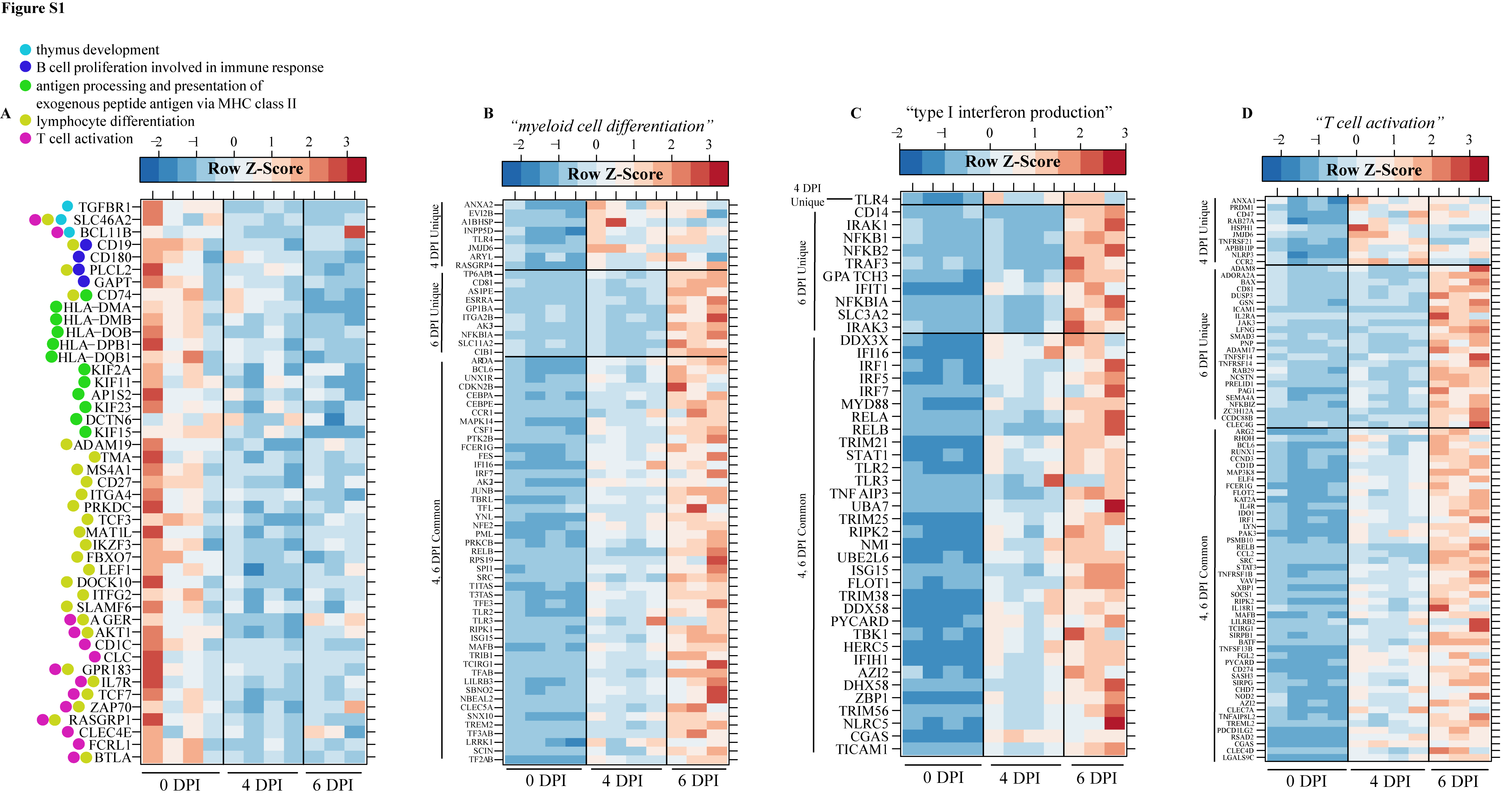

Supplement: Supplementary Figure 1 — Analysis of EBOV-Mayinga infection DEGs enriching to key GO terms. (A) Heatmap of downregulated genes enriching to “Thymus development,” “B cell proliferation involved in immune response,” “Antigen processing and presentation of exogenous peptide antigen via MHC class II,” “Lymphocyte differentiation,” and “T cell activation.” Membership of genes to gene ontology (GO) terms is denoted by matching colored marker. Heatmaps of 4 DPI unique, 6 DPI unique, or shared upregulated genes enriching to (B) “Myeloid cell differentiation,” (C) “T cell activation,” and (D) “Type I interferon production.” Each column represents one animal. Red presents upregulated; blue represents downregulated. Range of colors is based on scaled and centered rpkm values of the represented DEGs. [file Image_1.TIF]

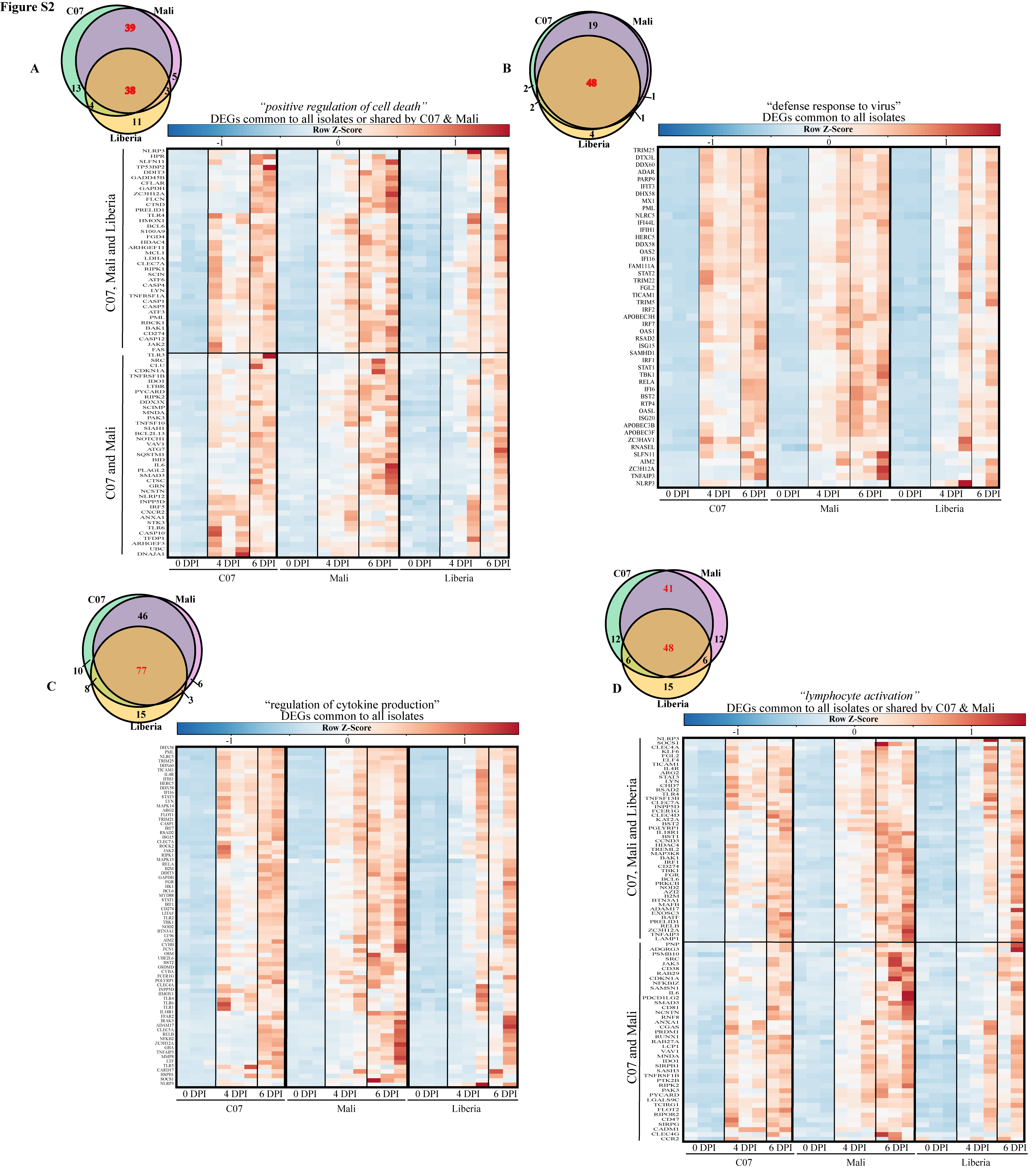

Supplement: Supplementary Figure 2 — Comparison of upregulated DEGs enriching to GO terms shared by early and late EBOV-Makona isolates. Heatmaps of shared upregulated DEGs enriching to GO terms common to all isolates: (A) “positive regulation of cell death,” (B) “defense response to virus,” (C) “regulation of cytokine production,” and (D) “lymphocyte activation.” Accompanying Venn diagrams depict the DEGs enriching to each isolate per given the given GO term. Only DEGs shared by either all isolates [e.g., panel (A)] or by Guinea C07 and Mali [e.g., panel (D)] are depicted and are indicated in red in the Venn diagram. Each column represents one animal. Red presents upregulated; blue represents downregulated. Range of colors is based on scaled and centered rpkm values of the represented DEGs. [file Image_2.TIF]

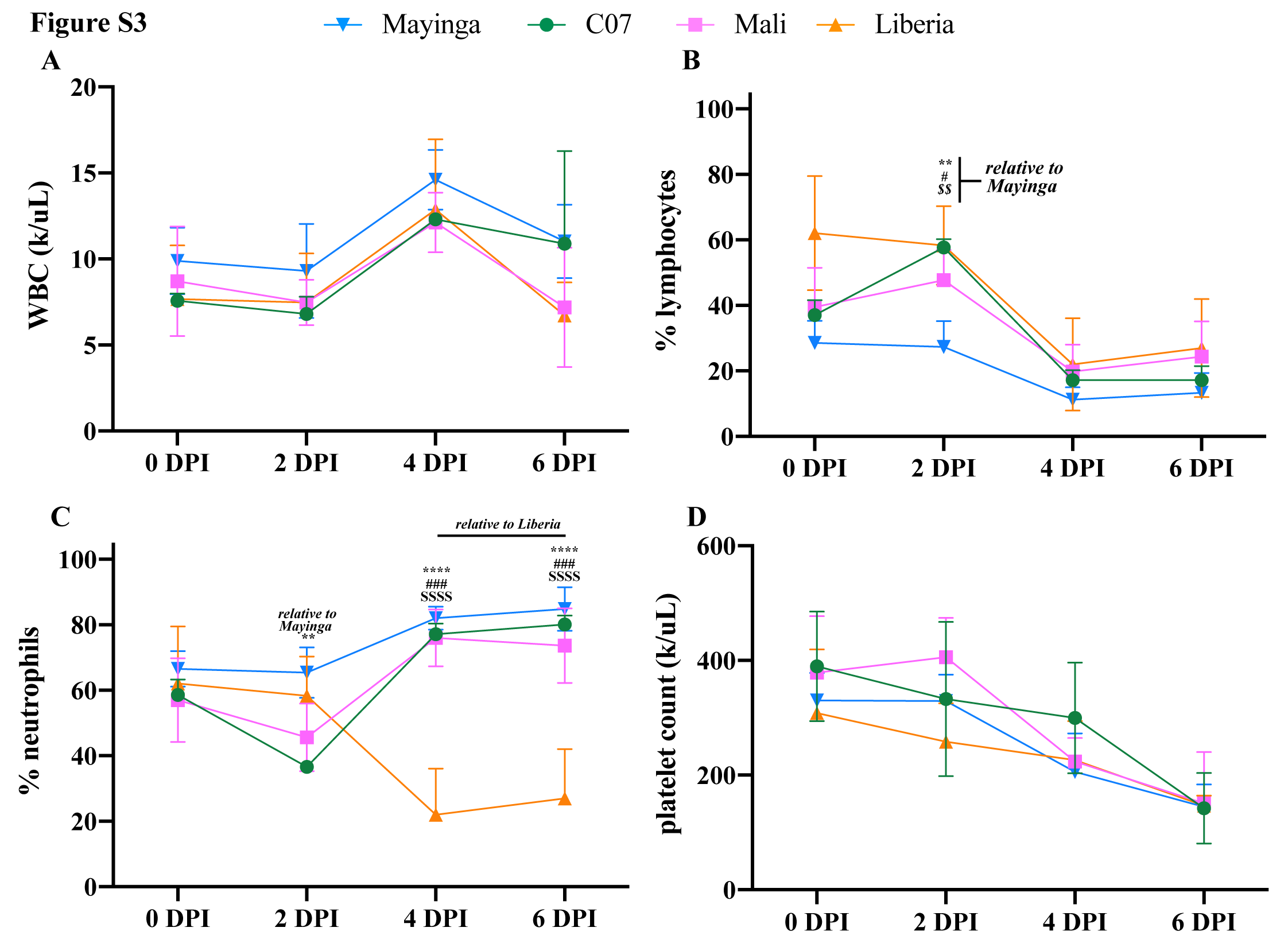

Supplement: Supplementary Figure 3 — Ebola virus infection results in lymphopenia, neutrophilia, and thrombocytopenia. (A) Total number of white blood cells (thousand/μl), (B) lymphocytes (% total cells), (C) neutrophils (%) total cells, and (D) platelet count (thousand/μl) for EBOV-Mayinga and EBOV-Makona isolates from d0 to d6. Significant differences between isolates at a given time point were determined with one-way ANOVA with multiple comparisons. ∗Guinea C07, #Mali, $Liberia. p-value < 0.05∗, p-value < 0.01∗∗, p-value < 0.001∗∗∗, and p-value < 0.0001****. [file Image_3.TIF]

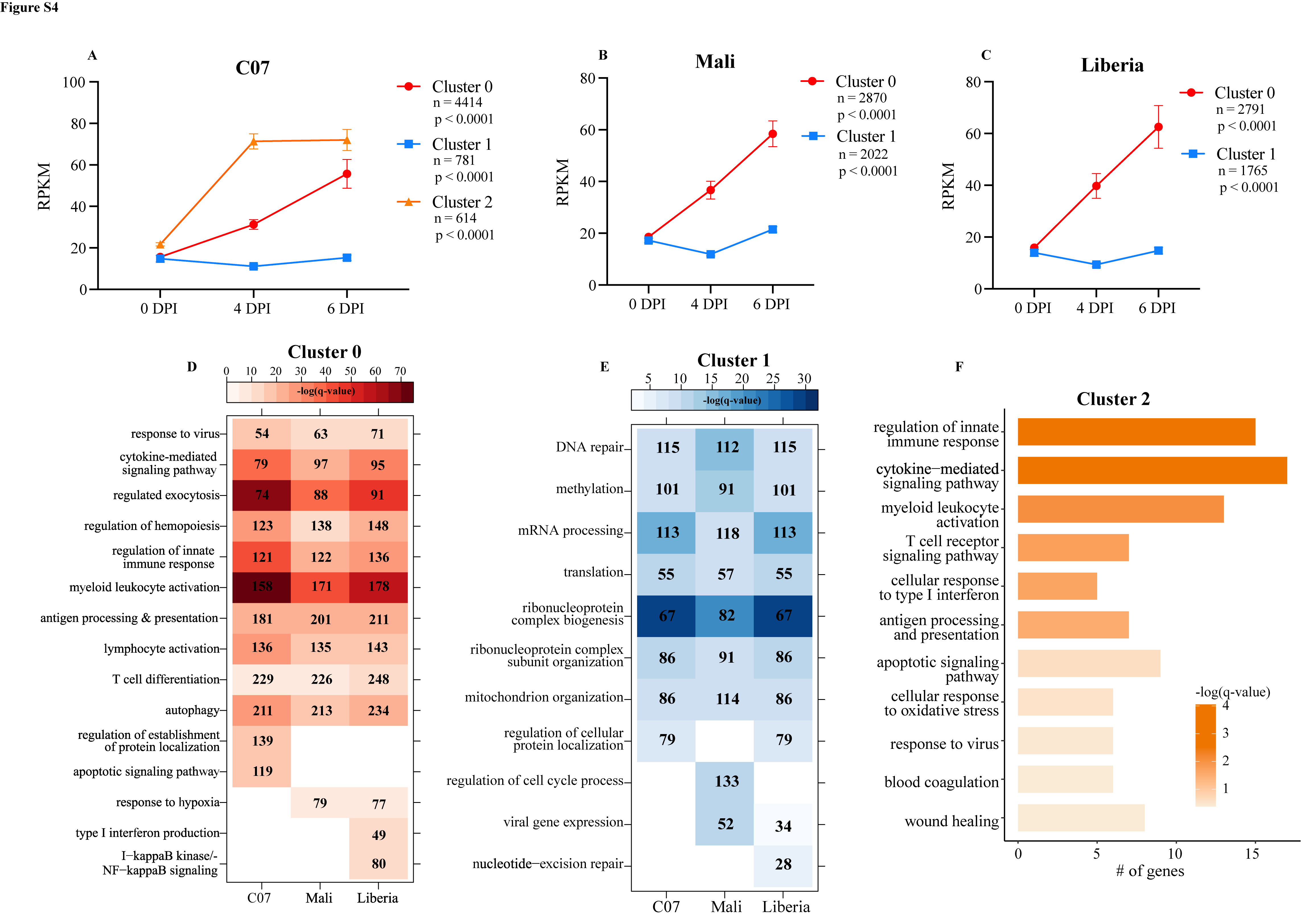

Supplement: Supplementary Figure 4 — STEM analysis of EBOV-Makona isolates. Gene expression graphs depicting the STEM clusters identified for EBOV-Makona (A) Guinea C07, (B) Mali, and (C) Liberia. GO term enrichment heatmaps for (D) cluster 0 and (E) cluster 1. Color intensity represents the statistical significance [shown as the −log(q-value)] with the range of colors based on GO terms with the lowest and highest significance statistic for the entire set of GO terms per cluster. The number of genes enriching to each GO term per column is represented in each box; white boxes indicate no statistical significance. (F) GO term bar graphs representing enrichment of genes in cluster 3. Horizontal bars represent the number of genes mapping to each GO term, while color intensity represents the −log(q-value) value of the corresponding GO term. Panels (D–F) follow the color key in plots A–C: cluster 0, red; cluster 1, blue; and cluster 2, orange. [file Image_4.TIF]

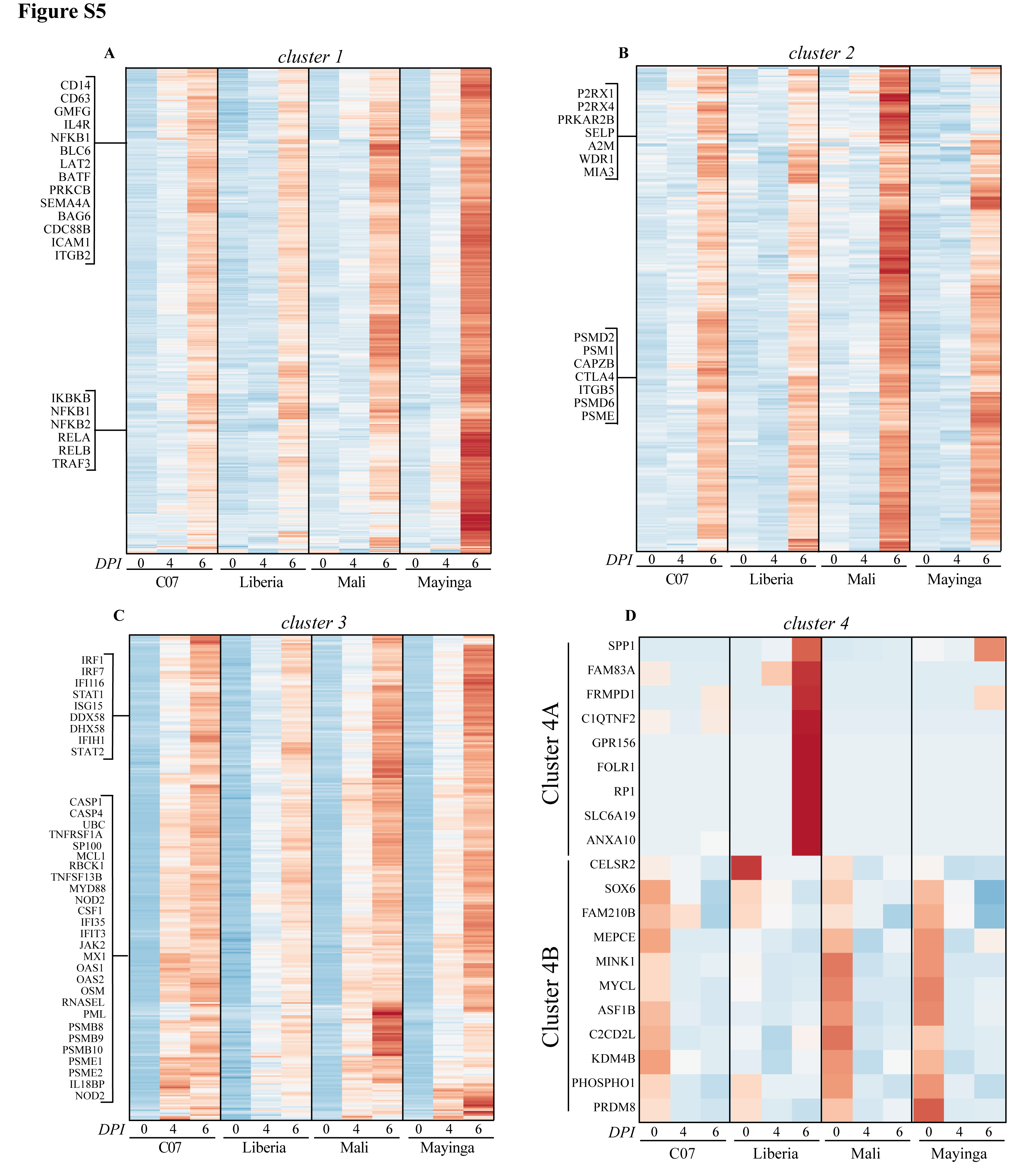

Supplement: Supplementary Figure 5 — Analysis of genes identified by MaSigPro, two-way forward regression analysis for EBOV-Makona and EBOV-Mayinga isolates. Heatmaps of genes identified by MaSigPro for (A) cluster 1, (B) cluster 2, (C) cluster 3, and (D) cluster 4. The latter was split into two sub-clusters: cluster 4A containing genes that were upregulated only in response to Liberia isolate and cluster 4B containing genes downregulated by all isolates. Select genes are indicated. Each column represents the median RPKM of all animals at a given day post infection. Red presents upregulated; blue represents downregulated. Range of colors is based on scaled and centered rpkm values of the represented DEGs. [file Image_5.TIF]
