# Supplementary material for: Transcriptional Analysis of Infection With Early or Late Isolates From the 2013–2016 West Africa Ebola Virus Epidemic Does Not Suggest Attenuated Pathogenicity as a Result of Genetic Variation
Source: Front Microbiol. 2021 Aug 13;12:714817. doi: 10.3389/fmicb.2021.714817 (PMC8415004; doi:10.3389/fmicb.2021.714817)
Supplement: Supplementary Table 1 — Statistical analysis (adjusted p-values) of longitudinal changes in cell and platelet populations. Adjusted p-values for data in Supplementary Figure 3. Two-way ANOVA with Geisser-Greenhouse correction to account for unequal variability of differences. Significant values highlighted in red. DPI, days post infection. [file Table_1.PDF]

Table S1. Statistical analysis (adjusted p-values) of longitudinal changes in cell and platelet populations.

	White blood cells				Lymphocytes				Neutrophil				Platelet			
	Mayinga	C07	Mali	Liberia	Mayinga	C07	Mali	Liberia	Mayinga	C07	Mali	Liberia	Mayinga	C07	Mali	Liberia
**0 DPI vs. 2 DPI**	0.986	0.772	0.845	0.998	0.905	0.063	0.512	0.986	0.998	0.035	0.464	0.960	>0.9999	0.299	0.707	0.448
**0 DPI vs. 4 DPI**	0.131	0.013	0.566	0.635	0.020	0.019	0.358	0.073	0.112	0.093	0.084	<0.0001	0.037	0.186	0.107	0.607
**0 DPI vs. 6 DPI**	0.917	0.771	0.963	0.984	0.074	0.136	0.242	0.065	0.046	0.040	0.154	0.000	0.000	0.022	0.030	0.266
**2 DPI vs. 4 DPI**	0.249	0.016	0.269	0.503	0.071	0.005	0.061	0.055	0.077	<0.0001	0.002	0.000	0.036	0.861	0.034	0.900
**2 DPI vs. 6 DPI**	0.685	0.537	1.000	0.985	0.202	0.016	0.003	0.110	0.030	<0.0001	0.005	0.001	0.019	0.131	0.051	0.219
**4 DPI vs. 6 DPI**	0.229	0.960	0.116	0.096	0.742	>0.9999	0.803	0.164	0.976	0.981	0.991	0.914	0.191	0.047	0.314	0.265

Adjusted p-values for data in Figure S3. Two-way ANOVA with Geisser-Greenhouse correction to account for unequal variability of differences. Significant values highlighted in red. DPI, days post infection.
